# The relationship between eHealth literacy and palliative care knowledge, attitudes, and practice among nurses: a cross-sectional study

**DOI:** 10.1186/s12912-023-01237-5

**Published:** 2023-03-21

**Authors:** Niu Yuan, Zhang-Hong Lv, Yuan-Yuan Wen, Chun-Rong Sun, Ting-Yu Tao, Dan Qian

**Affiliations:** 1 Department of Nursing, The First Affiliated Hospital of Zhejiang University School of Medicine, Hangzhou, China; 2 Department of Respiratory Medicine, The First Affiliated Hospital of Zhejiang University School of Medicine, Hangzhou, China; 3 Department of Surgical Oncology, The First Affiliated Hospital of Zhejiang University School of Medicine, Hangzhou, China; 4 Department of Ear Nose Throat, The First Affiliated Hospital of Zhejiang University School of Medicine, Hangzhou, China

**Keywords:** Nurses, eHealth literacy, Palliative care, Knowledge, attitudes, and practice

## Abstract

**Background:**

The crucial role that nurses play in offering palliative care to patients with life-threatening diseases is widely acknowledged, but the correlation between their eHealth literacy and their knowledge, attitudes, and practice in this domain has yet to be investigated. This study is conducted to investigate the status of eHealth literacy and knowledge, attitudes, and practice regarding palliative care among nurses, and to examine their relationship.

**Methods:**

A cross-sectional study design was conducted among 546 nurses selected from the first-class tertiary hospitals located both inside and outside of Zhejiang Province between May 12 and May 20, 2022. The online survey of eHealth literacy scale (eHEALS) and scale of knowledge, attitudes, and practice (KAP) regarding palliative care was performed using snowball sampling through the WeChat mini program “Questionnaire Star”. The Spearman rank correlation and binary logistic regression model were used to analyze the independent association between eHealth literacy and KAP toward palliative care.

**Results:**

The median scores of eHEALS and KAP regarding palliative care were 32 (interquartile range[IQR] 29 to 38) and 82 (IQR 54 to 106) points. The results of correlation analysis showed that the KAP regarding palliative care was significantly correlated with eHEALS (*rho* = 0.189, *P* < 0.001). In addition, the results of binary logistic regression analysis demonstrated that the eHEALS score was independently associated with the KAP score regarding palliative care when controlling for sociodemographic factors (*OR* = 2.109; *P* < 0.001).

**Conclusion:**

Nurses who worked in first-class tertiary hospitals have good levels of eHealth literacy, while the overall level of KAP regarding palliative care is moderate. Our findings highlight that the eHEALS score is independently associated with the KAP score regarding palliative care. Therefore, nursing managers should adopt multiple measures to comprehensively improve eHealth literacy among nurses, further enrich their knowledge of palliative care, promote a positive transformation of attitudes towards palliative care, and efficiently implement palliative care practice, in order to promote high-quality development of palliative care.

## Introduction

In the context of the ever-accelerating global digital development, Internet information continues to profoundly influence the public’s work and daily life, and people are increasingly dependent on the Internet to access health-related information [1]. However, the content and sources of health-related information on the Internet are complex and diverse. The dissemination of false information can have adverse consequences in the field of health, such as causing public panic over infectious diseases, preventing or delaying effective medical care, and even posing a threat to lives and health [2–4]. Without the ability to access, identify, and effectively use Internet resources, healthcare professionals may suffer from reduced clinical and research capabilities, ultimately hindering the development of the medical and healthcare industry. Electronic Health literacy was first proposed by Norman and Skinner in 2006 [5]. It refers to the ability of individuals to access, understand and evaluate health information from digital resources, and to effectively utilize health-related information on the Internet, which could be measured by eHEALS. With the rapid and high-quality development of the digital medical industry in China, a Chinese version of eHEALS (C-eHEALS) has been developed by Guo et al. [6] and compared with other eHEALS findings [7], which suggested that C-eHEALS has good reliability and validity. As the main force of health promotion, nurses are increasingly using the Internet and related technologies to manage patients’ health concerns [8]. How nursing staff can quickly obtain accurate health-related information in the busy medical system is directly related to their ability to effectively solve clinical problems, provide advanced medical care services to patients, share decision-making, and implement behaviors that promote health and prevent disease.

Palliative care refers to a comprehensive care model that focuses on improving the quality of life of patients with malignant tumors or incurable diseases and their families, mainly by alleviating symptoms, reducing suffering, and addressing physical, psychological, and social issues in end-of-life care. The goal is to help patients and their families face death properly [9]. In the 21st century, the incidence of diseases, such as malignant tumors and AIDS remains high [10, 11]. In recent years, the global outbreaks of COVID-19 [12], combined with the transformation of the traditional medical models and the increasingly serious trend of aging populations, make it imperative to provide palliative care to terminally ill patients and their families and to improve the quality of end-of-life care [13]. A cross-sectional survey carried out by Knapp et al. [14] stated that parents of children suffering life-threatening illnesses with high-level eHealth literacy were more likely to search the internet rather than consult a doctor. In addition, the providers of palliative care held the belief that digital health has the potential to enhance the quality of palliative care [15]. However, it has not yet been studied whether the eHealth literacy of nursing staff will have an impact on this potential improvement.

Currently, there is a certain gap between China’s palliative care and developed countries around the world. A previous study has shown that, apart from the influence of Chinese traditional culture and economy, a significant factor contributing to the lack of palliative care in China is the low level of knowledge, attitudes, and practice (KAP) of palliative care in this area [16]. Inadequate awareness and understanding of palliative care, as well as a lack of corresponding knowledge and skills, limit the implementation of palliative care [17, 18]. In order to effectively assess the KAP related to palliative care among nurses, the knowledge and attitudes questionnaires were translated into Chinese by Zou, et al. [19], and the practice questionnaire was developed by Yang, et al. [20], which has been widely applied for nursing studies [21, 22]. How to quickly and efficiently improve the level of KAP regarding palliative care among nursing staff, and promote the development of palliative care in China, is worth our deep consideration. During the COVID-19 pandemic, lower levels of eHealth literacy were independently associated with less protective KAP in the field of coronavirus among Chinese and American adults [23, 24]. However, whether eHealth literacy has a significant independent association with KAP related to palliative care among nurses still requires further investigation. Therefore, the purpose of this study was to assess eHealth literacy and palliative care KAP levels among the nursing staff in first-class tertiary hospitals located both inside and outside Zhejiang Province, and to analyze their relationship.

## Methods

### Study design and setting

A cross-sectional survey was conducted using the WeChat mini program “Questionnaire Star”, which involved nursing staff working in first-class tertiary hospitals located both inside and outside Zhejiang Province between May 12 and May 20, 2022.

### Study population and sampling

A snowball sampling method was used to recruit nursing staff through the WeChat mini program “Questionnaire Star”. In this process, the nurses who worked in the Department of Respiratory Medicine, Surgical Oncology, and Ear Nose Throat of The First Affiliated Hospital of Zhejiang University School of Medicine were initially invited to complete the questionnaire. In addition, other nurses from a total of 27 first-class tertiary hospitals were recruited from the invitation of initial respondents via WeChat contacts. To be eligible for the survey, nurses had to be (1) worked at the clinical frontline holding a registered nurse’s license, and (2) informed and willing to participate in this study. The exclusion criteria included (1) resigned nurses or (2) administrative nurses who were not engaged in direct patient care. There were 561 nurses from Zhejiang, Guangdong, Hubei, and Anhui provinces in China who clicked the web link of the questionnaire. After deleting the multiple responses of same IP address and short completion time (under 300 s), there were 546 valid questionnaire in total (response rate: 97.3%). This study was approved by the Ethics Committee of The First Affiliated Hospital of Zhejiang University School of Medicine, and informed consent was obtained from all nurses before their voluntary participation.

### Study instruments

Three questionnaires were utilized in this study to collect sociodemographic data and measure eHealth literacy and KAP regarding palliative care.

#### Sociodemographic questionnaire

Respondents provided data on gender (women or men), age, education [college, bachelor, master or doctor of philosophy (Ph.D.)], job titles (nurse, senior nurse, supervisor nurse, co-chief superintendent nurse or chief superintendent nurse), work experience, English proficiency (below CET-4, CET-4, CET-6 or above CET-6), computer proficiency (NCRE Grade 1, 2, 3 or 4), whether receiving training in palliative care (Yes or not), and number of end-of-life patients cared for.

#### C-eHEALS questionnaire

The eHEALS was developed by Norman, et al. [5], and translated into Chinese by Guo, et al. [6], which is primarily used to evaluate individuals’ ability to use electronic health information to address health issues. This scale (Cronbach’s α = 0.913) consists of 8 items in total that measure three dimensions as follows: application ability (5 items), judgment ability (2 items), and decision-making ability (1 item) to online health information and services [6]. Each C-eHEALS item is scored using the Likert 5-level scoring method, and the total score is the sum of each item. From “very inconsistent” to “very consistent”, 1 to 5 points were counted, with a total score of 8 to 40. The respondents with higher scores indicate high-level eHealth literacy skills.

#### KAP questionnaire towards palliative care

The KAP questionnaire about palliative care includes three parts: knowledge (20 items), attitudes (12 items), and practice (8 items). The first part is about knowledge which scores according to ‘right’/‘wrong’ or ‘unclear’ feedback; 1 point for right and 0 points for wrong or unclear. The other two parts are both scored using a Likert 5-level scoring method ranging from 1 to 5. The total score of each part is the sum of all individual scores, and the overall score of the KAP scale is the sum of the total scores of the three parts. The higher the score, the more knowledgeable (score ranges from 0 to 20), favorable (score ranges from 12 to 60), and proactive (score ranges from 8 to 40) toward palliative care of respondents.

The knowledge and attitudes parts were developed by Ross, et al. [25] and Bradley, et al. [26] respectively, and translated into Chinese by Zou, et al. [19]. The practice part was developed by Yang, et al. [20]. The first part of the KAP questionnaire (Cronbach’s *α* = 0.758) concerns palliative care nurses’ knowledge about principle and philosophy of palliative care (4 items), pain and symptom management (13 items), and psychosocial and spiritual support (3 items) [19]. The second part (Cronbach’s *α* = 0.794) concerns palliative care nurses’ attitudes toward professional roles and responsibilities (4 items), effectiveness of palliative care (5 items), and nurse-patient communication (3 items) [19]. The third part (Cronbach’s *α* = 0.910) concerns palliative care nurses’ practice which included physical care (4 items), psychological care (2 items), and social care (2 items) [20].

### Statistical analysis

Statistical analysis was performed using SPSS (the Statistical Package for the Social Sciences) software ver. 25 and R software (version 4.1.0). The Shapiro-Wilk test was used to assess the normality of age, work experience, eHEALS score, and scores in the three parts of the KAP questionnaire. The results of the Shapiro-Wilk test showed that *P* < 0.05, which indicated these data did not have a normal distribution and expressed as median (interquartile range, IQR). Given the KAP scores regarding palliative care are discrete in nature, they were grouped into 2 ordinal levels using the median score as the threshold [27]. This approach was adopted to indicate significant increases in the levels of the outcomes.

We used R software to calculate the Spearman rank correlation coefficient (Spearman *ρ*) and perform a correlation test (cor. test) between eHEALS scores and the total scores of KAP regarding palliative care, as well as its three individual parts. In comparing the differences in the high and low-level KAP of palliative care between nursing staff, the Wilcoxon rank sum test was used to compare the differences in age and working experience, while the chi-square test was used to analyze the differences in other count data. According to the results of the single factor analysis, a binary logistic regression model was constructed to assess the association of KAP related to palliative care with eHealth literacy using the “glm” function in the R language, and the *P*, *OR*, and 95%*CI* for each included variable were recorded. *P* < 0.05 was considered to be statistically significant.

## Results

### Participants

A total of 546 nursing staff participated in the survey, of which women accounted for 97.25% (n = 531). The age group of 20 to 35 years old was the most common, accounting for 71.98% (n = 393). Most of them had a bachelor’s degree, accounting for 91.45% (n = 503). The job titles are mostly senior nurse and supervisor nurse, accounting for 42.73% (n = 235) and 38.73% (n = 213), respectively. The majority had 8 to 10 years of work experience, accounting for 25.27% (n = 138). The most common English proficiency level was level 4, accounting for 38.73% (n = 213), and the most common computer proficiency level was grade 2, accounting for 57.82% (n = 318).

### eHealth literacy and KAP regarding palliative care among nurses

**Fig. 1:**
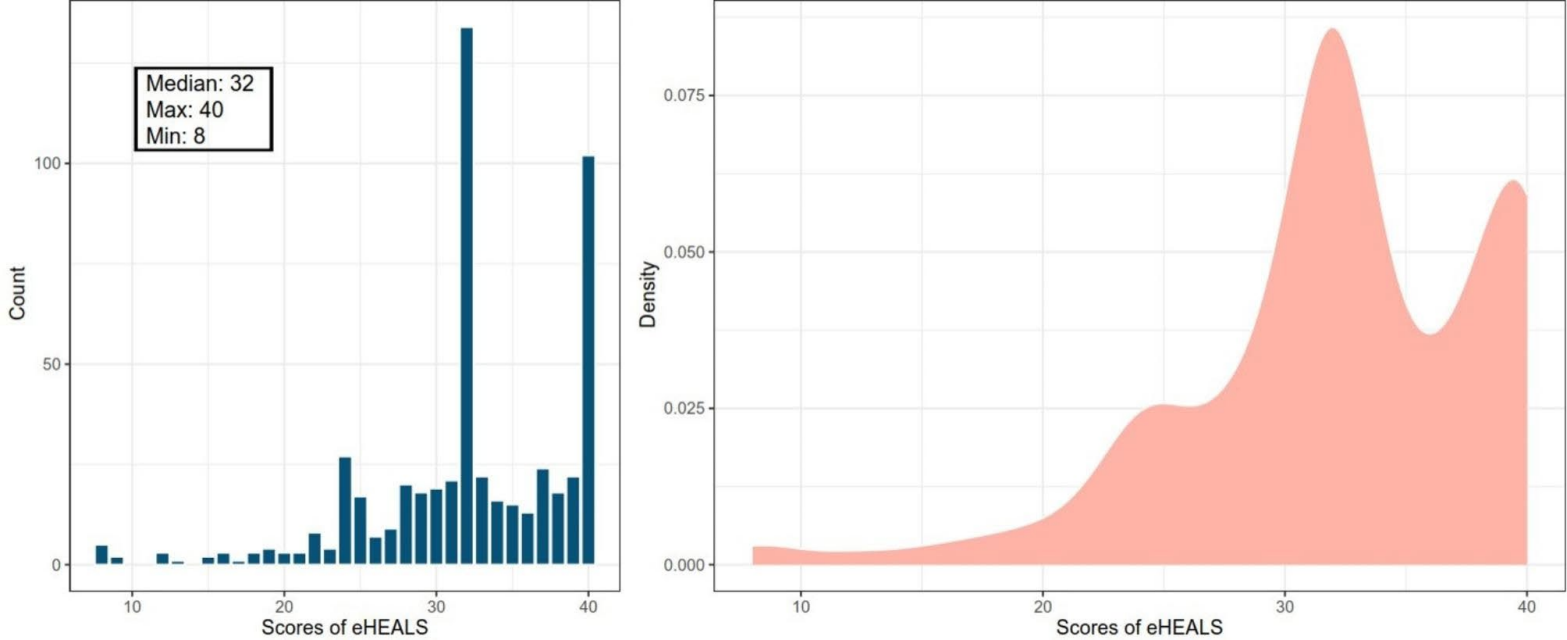
The distribution of eHEALS scores among 546 nurses;

**Fig. 2:**
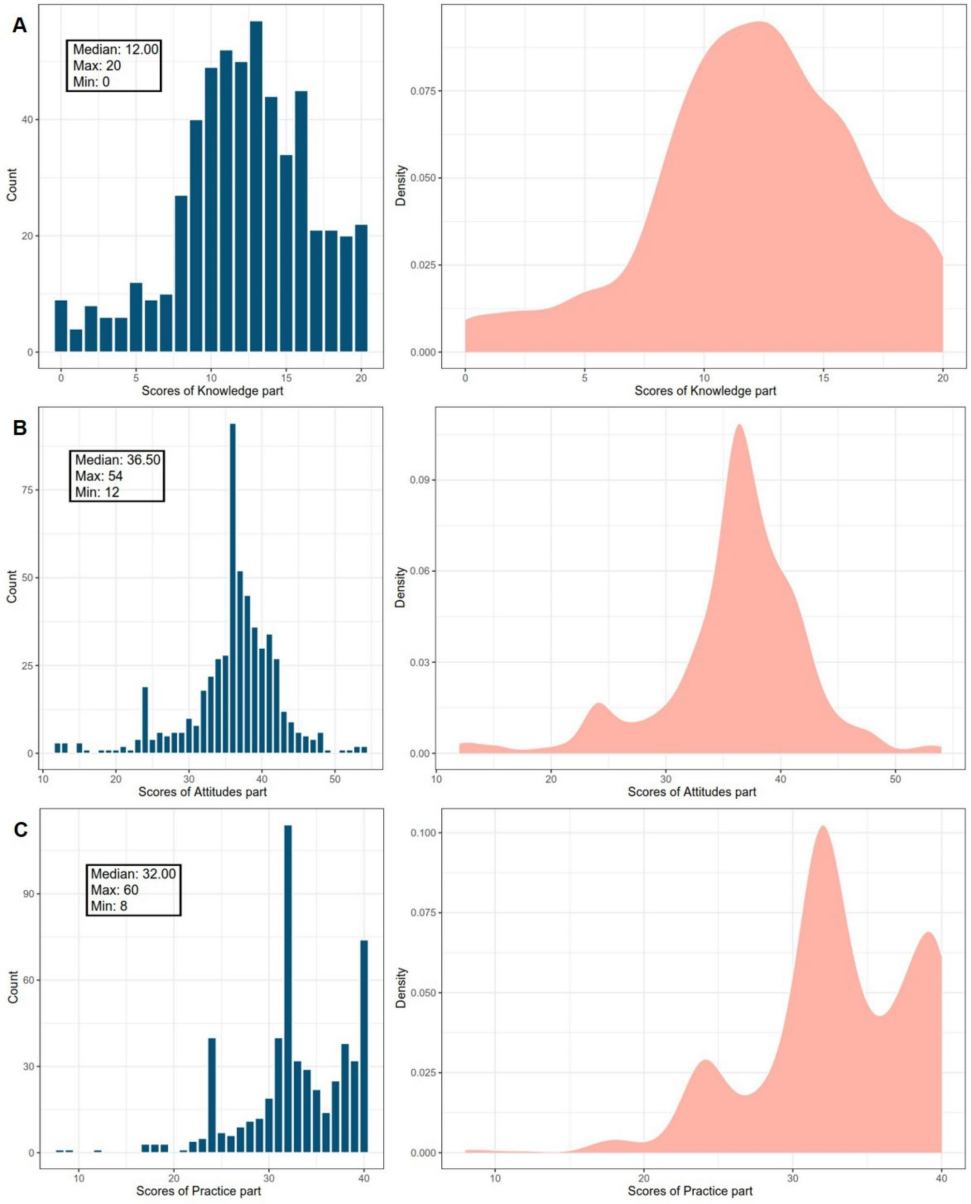
The distribution of KAP scores among 546 nurses; A: Knowledge scores; B: Attitudes scores; C: Practice scores

The eHEALS score of 546 nursing staff was 32 (IQR 29 to 38) points. The lowest and highest eHEALS scores were 8 points (n = 5, 0.92%) and 40 points (n = 102, 18.68%), respectively (Fig. 1). The proportion of nurses who responded “very consistent” to the term “I am very confident in making health-related decisions based on online information” was the lowest among all responses for 8 items. The total score range for the KAP questionnaire regarding palliative care among nurses was 54 to 106, with a median total score of 82 (IQR 76 to 87). The knowledge, attitudes, and practice part scores ranged from 0 to 20, 12 to 54, and 8 to 40, respectively, with median scores of 12 (IQR 10 to 15), 36.5 (IQR 34 to 40), and 32 (IQR 31 to 38), respectively (Fig. 2).

### Correlation analysis between eHealth literacy and KAP regarding palliative care

The results of the correlation analysis showed that the Spearman rank correlation coefficient (*rho*) between eHEALS and the overall score of KAP related to palliative care was 0.189, with a *P*-value of 8.89e-06. The rho values between eHEALS and knowledge, attitudes, and practice part were 0.130, -6.622e-03, and 0.262, respectively, and the corresponding *P*-value were 2.381e-03, 0.877, and 4.77e-10, respectively.

### Factors independently associated with KAP regarding palliative care

Taking the median score of KAP regarding palliative care as the threshold, the nurses were divided into high and low-level groups. Single factor and binary logistic regression analysis were conducted on the sociodemographic data and eHEALS score as independent variables for KAP regarding palliative care. The results of the single-factor analysis showed that there were statistically significant differences in job titles, the number of end-of-life patients cared for, and the eHEALS scores (all *P* < 0.05) (Table 1). The results of logistic regression analysis showed that eHEALS score was independently associated with the KAP score regarding palliative care when controlling for sociodemographic factors (*OR* = 2.109; *P* < 0.001) (Table 2).

**Table 1:** Single factor analysis of KAP levels regarding palliative care among nurses

Variables	Low-level KAP (*n* = 288)	High-level KAP (*n* = 258)	*P*-value
**Age**	31 (28, 54)	32 (27, 51)	0.138
**Education**			0.505
College	21 (7.29%)	17 (6.59%)	
Bachelor	262 (90.97%)	238 (92.25%)	
Master	4 (1.39%)	3 (1.16%)	
Ph.D.	1 (0.35%)	0 (0%)	
**Job titles**			0.038
Nurse	36 (12.50%)	30 (11.63%)	
Senior nurse	137 (47.57%)	98 (37.98%)	
Supervisor nurse	104 (36.11%)	107 (41.47%)	
Co-chief superintendent nurse	11 (3.82%)	22 (8.53%)	
Chief superintendent nurse	0 (0%)	1 (0.39)	
**Work experience**	9 (5, 12)	10 (5, 15)	0.159
**English proficiency**			0.593
Below CET-4	145 (50.35%)	119 (46.12%)	
CET-4	106 (36.80%)	105 (40.70%)	
CET-6	37 (12.85%)	34 (13.18%)	
Above CET-6	0 (0%)	0 (0%)	
**Computer proficiency**			0.417
NCRE Grade 1	102 (35.42%)	90 (34.89%)	
NCRE Grade 2	160 (55.56%)	154 (59.69%)	
NCRE Grade 3	20 (6.94%)	11 (4.26%)	
NCRE Grade 4	6 (2.08%)	3 (1.16%)	
**Whether receiving training in palliative care**			0.191
Yes	57 (19.79%)	64 (24.81%)	
No	231 (80.21%)	194 (75.19%)	
**Number of end-of-life patients cared for**			0.048
< 4	121 (39.81%)	92 (37.93%)	
4–19	84 (27.39%)	66 (27.59%)	
≥ 20	83 (32.80%)	100 (34.48%)	
**eHEALS score**	32 (28, 36)	33 (31, 39)	2.555e-06

**Table 2:** Binary logistic regression analysis of KAP regarding palliative care

Variables	*Univariate*			*Multivariate*		
	*OR*	95%*CI*	*P*-value	*OR*	95%*CI*	*P*-value
Age	1.029	1.002–1.057	0.0344	0.999	0.914–1.086	0.990
Education	1.019	0.526–1.988	0.956	1.783	0.832–3.869	0.138
Job title	1.359	1.094–1.696	0.006	1.416	0.941–2.143	0.097
Work experience	1.026	1.001–1.053	0.041	1.004	0.924–1.096	0.9122
English proficiency	1.124	0.883–1.431	0.343	1.323	0.980–1.793	0.069
Computer proficiency	0.924	0.709–1.203	0.558	0.783	0.583–1.047	0.102
Whether receiving training in palliative care	1.341	0.893–2.023	0.159	1.122	0.723–1.747	0.609
Number of end-of-life patients cared for	1.222	1.002–1.492	0.048	1.168	0.938–1.454	0.165
eHEALS Score	2.008	1.423–2.844	7.910e-05	2.109	1.464–3.053	9.830e-05

## Discussion

With the continuous development of global Internet healthcare and the increasing demand for health among people, nurses are also compelled to continuously improve their comprehensive quality, particularly their eHealth literacy. This will enable them to make the correct choices in disease prevention, health promotion, and clinical nursing practice. The results of this study showed that the median eHEALS score among 546 nursing staff from 28 first-class tertiary hospitals in China was 32 (IQR 29 to 38) points, indicating a good level of e-health literacy. Kritsotakis, et al. [28] investigated the eHealth literacy levels of 200 Greek nurses through a cross-sectional study design and found that the average eHEALS score was 30.7 ± 5.8, which is consistent with the results of this study. However, this study found that there were still 5 nurses with eHEALS scores as low as 8 points, which is lower than that of nursing undergraduates both in China and abroad [29, 30], indicating some nursing staff have poor abilities to access and understand health-related information and to solve health problems through electronic media, and there is still much room for improvement. The proportion of nurses who responded “very consistent” to the term “I am very confident in making health-related decisions based on online information” was the lowest among all responses for 8 items, indicating that nursing staff lack confidence in using electronic media to obtain and understand health-related information to address health concerns, which indirectly reflects their weak evidence-based nursing skills. Song, et al. [31] conducted a cross-sectional study and found that the eHealth literacy level of family members of newly diagnosed localized prostate cancer patients determined their level of participation in clinical decision-making. In clinical nursing practice, nurses should make full use of Internet resources to search, discover, understand, and apply critical thinking to seek evidence-based solutions to difficult nursing problems, in order to provide better quality nursing services for patients [32].

This study also investigated the levels of KAP regarding palliative care among nurses and found that the overall level of palliative care KAP among 546 nurses was moderate of 82 (IQR 54 to 106) points, which was consistent with the results of another domestic study [33]. Findings from a systematic review indicated a strong necessity for enhancing the understanding and execution of evidence-based practice among community nurses, despite their positive attitudes towards it [34]. Fu, et al.[35] summarized the current status of palliative care for patients with advanced COPD, and pointed out that the development of palliative care urgently needs the participation of specialized nurses with rich knowledge and experience. Therefore, nursing managers should take comprehensive measures to provide nursing staff with end-of-life care knowledge, as well as training and education in physiology, psychology, social support, laws and regulations, and other aspects, such as establishing an independent discipline of palliative care, constructing a palliative care information sharing platform [36], strengthening on-the-job training for palliative care, promoting appropriate technologies related to palliative care [37], and home-based remote palliative care, etc. As for the nursing staff themselves, they should continuously tap into their own inner potential and keep learning, so as to transform their cognition and attitudes toward palliative care, and effectively implement palliative care practice.

The KAP regarding palliative care has found to be positively correlated with eHEALS (rho = 0.189, P < 0.001). This means that nursing staff with higher eHealth literacy tend to have higher levels of KAP in palliative care, which is consistent with previous research findings [38]. People with high-level eHealth literacy are generally better at solving health problems and promoting healthy behaviors than those with low-level eHealth literacy. In the clinical practice of palliative care, nursing staff may encounter challenging problems or lack relevant nursing experience. Nursing staff with high e-health literacy have the ability to obtain, identify and utilize internet resources efficiently, thereby effectively solving the practical clinical problems of palliative care. In our study, we demonstrated that the eHEALS score was independently associated with the KAP score regarding palliative care when controlling for sociodemographic factors (OR = 2.109; P < 0.001). By igniting the intrinsic motivation of nurses, effective and comprehensive palliative care be provided for patients, and the development and progress of palliative care in China can be improved.

### Limitations

There were several limitations existed in our study. Firstly, the use of snowball sampling, a non-probability sampling method, made it difficult to assess the reliability of the results through statistical tests. However, we attempted to improve the credibility of our research findings by performing a binary logistic regression model. Secondly, the number of respondents size from outside of Zhejiang Province was small. Finally, our study was conducted using the WeChat mini program “Questionnaire Star”, which could have introduced a bias towards nurses who are more technologically and internet-savvy.

## Conclusion

Nursing staff in first-class tertiary hospitals have a good level of eHealth literacy, but the overall level of KAP regarding palliative care is moderate. There is a positive correlation and an independent association between KAP related to palliative and eHealth literacy. Therefore, nursing managers should adopt various measures to comprehensively improve the eHealth literacy level of nursing staff, further enrich the knowledge of palliative care, promote positive attitudes transformation, and efficiently implement palliative care practices to promote high-quality development of palliative care.

## Data Availability

The data that support our findings are available from the corresponding author, [NY], upon reasonable request.
